# Work Sustainability Among Male Cancer Survivors After Returning to Work

**DOI:** 10.2188/jea.JE20160152

**Published:** 2018-02-05

**Authors:** Motoki Endo, Yasuo Haruyama, Go Muto, Kosuke Kiyohara, Tetsuya Mizoue, Noriko Kojimahara, Naohito Yamaguchi

**Affiliations:** 1 Department of Public Health, Tokyo Women’s Medical University, Tokyo, Japan; 2 Department of Public Health, Dokkyo Medical University, School of Medicine, Mibu, Tochigi, Japan; 3 Department of Epidemiology and Environmental Health, Juntendo University Faculty of Medicine, Tokyo, Japan; 4 Department of Epidemiology and Prevention, Center for Clinical Sciences, National Center for Global Health and Medicine, Tokyo, Japan

**Keywords:** work sustainability, cancer survivors, return to work (RTW), work continuance rate, sick leave

## Abstract

**Background:**

Few studies have investigated the work continuance rate among cancer survivors after return to work (RTW). The objective of this study was to clarify work sustainability after RTW among Japanese male cancer survivors.

**Methods:**

We collected data on male cancer survivors from an occupational health register. Inclusion criteria were as follows: employees who returned to work after an episode of sick leave due to clinically certified cancer diagnosed between January 1, 2000 and December 31, 2011.

**Results:**

Of 1,033 male employees who were diagnosed with cancer, 786 employees (76.1%) returned to work after their first episode of sick leave due to cancer. Work continuance rates among all subjects were 80.1% 1 year after RTW and 48.5% 5 years after RTW. The mean duration of work after RTW was 4.5 years. The work continuance rates varied significantly by cancer type. The “Lung” and “Hepatic, Pancreatic” cancer groups had the shortest duration of work (0.9 year after RTW).

**Conclusions:**

Of workers who returned to work after their first episode of leave after cancer, 50% continued to work after 5 years in large-scale companies. There was a steep decrease in work continuance rates during the first year after RTW, with considerable differences according to cancer site.

## INTRODUCTION

In developed countries, as the population ages with increasing cancer survival rates, the proportion of working-age cancer survivors is expected to increase.^1^^–^^4^ In Japan, the Japanese Cancer Surveillance Research Group reported that about 30% of all diagnosed cancer patients were of working age (20 to 60 years of age) in 2010.^5^ Several previous studies have conducted follow-up for employees who returned to work after sick leave due to cancer in Japan.^2^^,^^6^ While many types of cancer have become more like other chronic diseases, cancer still remains a life-threatening disease.^7^^,^^8^ In many cases, cancer greatly influences health status, mental health, and overall quality of life.^3^

Paid employment is a particularly important aspect of life for cancer survivors of working age, and return to work (RTW) of cancer survivors is closely associated with not only the individual, but also employers and society.^3^^,^^9^^–^^12^ The ability to work among cancer survivors is said to be very complex, due to various medical and non-medical factors.^10^ RTW rates seem to be greatly affected by personal factors, work related factors, and social factors, with previous studies reporting RTW rates among cancer survivors ranging from 44% to 100%.^2^^,^^13^^,^^14^

Maintaining employment after RTW can be challenging for cancer survivors, especially with regard to physical and mental health.^12^^,^^15^^,^^16^ After RTW, cancer survivors are often confronted with great difficulties, which can be disease-related (eg, burdensome treatment or advanced stages of the disease), physical (eg, fatigue, pain, or nausea), and work related (eg, physical demands at work or difficult relationships with superiors and colleagues).^13^ Previous studies have shown that most cancer survivors are employed, but work sustainability (both physical and mental work ability) can fail due to cancer-related reasons.^12^^,^^17^ Many studies of cancer survivors reported that they were more likely to lose their jobs than cancer-free individuals.^18^^–^^20^ Amir et al reported that the work adjustments provided after RTW could be very important for cancer survivors.^9^ In 2016, the Japanese Ministry of Health, Labour and Welfare published guidelines for the support of therapy and working life in the Japanese workforce, similar to guidelines published in the Netherlands and the United Kingdom.^6^^,^^21^^,^^22^

However, there is little evidence of the potential impact of cancer, its treatment, and long-term side effects on work.^3^^,^^12^ Despite the importance of this subject, no large-scale workforce-based study has been conducted to clarify the work continuance rate after RTW among cancer survivors, stratified by cancer site. We hypothesized that, in large-scaled companies, the work continuance rate among cancer survivors was high. The objective of this study was to clarify the course and predictors of the time to failure of work sustainability among male cancer survivors, stratified by cancer site.

## METHODS

### Study design and the process of RTW

This was a retrospective cohort study of the course after RTW among Japanese cancer survivors working in large-scale companies. Registered sick leave data was obtained from a private occupational health center. The occupational health center contracted occupational physicians (OPs) to provide employees who belong to a large-scale Japanese company group that includes various companies (eg, telecommunications, logistics, energy, and construction), as described in a previous study.^2^ About 68,000 employees were working for these companies on a full-time basis from 2000 through 2011.

Sick leave due to cancer is accepted with a physician’s certificate stating that ‘this employee cannot work due to cancer’. Employees with cancer submit the certificate to the human resources department (HR). The OP confirms the medical validity of the physician issued certificate (sent from HR) and the certificate is returned to the HR department. Then, the OP records the cause of sick leave, referring to the World Health Organization’s 10^th^ International Classification of Diseases (ICD-10).

To RTW, an employee with cancer is required to submit a physician’s certificate stating that ‘this person can return to work’ to HR, and undergo an interview with an OP for further confirmation that RTW is medically acceptable. The company judges whether it should allow the employee to RTW, based on the physician’s certificate, the OP interview, and the intention of the company. If the company allows the employee’s RTW, the OP issues a certificate describing appropriate work schedules, such as full-time or part-time or the amount of overtime that can be worked per month. After RTW, the OP interviews the employee regularly, once or twice per month.

Likewise, recurrent sick leave after RTW is also accepted only with a physician’s certificate stating that ‘this person cannot work due to cancer’. We also collected data on episodes of recurrent sick leave, even if the sick leave occurred the day after RTW, based on a physician’s certificate. Therefore, the only data collected for this study were based on physicians’ certificates.

### Subjects and the inclusion criteria

Subjects were selected from the health register, in a search of all employees who satisfied the inclusion criteria.

Inclusion criteria were: male employees registered in the Health Data System, aged 15 to 60 years, who returned to work after the first sick leave due to cancer (C01–C99; ICD-10, based on a physician’s certificate, between January 1, 2000, and December 31, 2011).

The subjects of this study were employees who returned to work after an episode of sick leave due to clinically certified cancer diagnosed between January 1, 2000 and December 31, 2011. Of the cancer survivors, 786 employees returned to work.

Based on these inclusion criteria, the first sick leave was not a recurrence, because employees who had had previous episodes of sick leave due to cancer before December 31, 1999 were not included in the study.

### Ethics

This study was approved by the Medical Ethics Committee of Tokyo Women’s Medical University (number: 3244).

### Statistical analysis

The period of sustained work without recurrent sick leave or resignation was expressed using a Kaplan-Meier survival analysis curve. The person-days were calculated based on the follow-up period. The day used to measure the beginning of the duration of this study was the first day of RTW after sick leave due to cancer. The censored day for the duration of this study was either the end of follow-up (December 31, 2011) or the day of retirement (March 31 of the year that a subject becomes 60 years old), whichever came first. The definition of the event day for this analysis were the first day of recurrent sick leave due to any illness certified by physicians or the day of resignation before retirement (60 years old). The day that a subject died was regarded as a day of recurrent sick leave. Maximum and median duration of follow-up were 12.3 years and 4.5 years, respectively.

To analyze the risk factors for failure of work sustainability, hazard ratios (HR) and their 95% confidence interval (CI) were analyzed using IBM SPSS Statistics for Windows (version 23.0; IBM Corp, Armonk, NY, USA). Age, duration of sick leave, cancer site, calendar year (year of sickness leave), manager, and job title were chosen as risk factors for failure of work sustainability. Subjects were stratified into four categorized age groups by quartiles: 48 years or younger (reference), 49–52 years, 53–56 years, and 57 years or older. Subjects were stratified into three categorized sick-leave groups: 60 days or less (reference), 61–120 days, and 121 days or more. We used seven cancer sites: “Gastric” (reference), “Esophageal”, “Intestinal”, “Lung”, “Hepatic, Pancreatic”, “Male Genital”, “Urinary”, and “Blood”. Subjects were stratified by the year of sick leave into three categorized calendar-year groups: 2000–2003 (reference), 2004–2007, and 2008–2011. A ‘manager’ was defined as an individual who belonged to an administrative post, which in Japanese organizations is considered to be a position higher than a section chief. Job title was divided into two groups: ‘desk worker’ (for example, ‘office worker’, ‘sales worker’, ‘researcher’), which involves a mainly mental workload, and ‘manual worker’ (for example, ‘technician’), which involves a mainly physical workload. By analyzing relationships among continuous variables, we determined *P*-values for trends in age and sick leave.

The Cox model assumes that the hazard ratio remains constant over time. We virtually checked the log-minus-log graphs to test this assumption and found no indication of any violation. We performed single variable analyses and multivariable analyses including all variables. Trend associations for age and duration of sick leave were assessed by assigning the median of each exposure category and modeling this as a continuous variable.

## RESULTS

Of the 1,033 cancer survivors identified in the register who were diagnosed between January 2000 and December 2011, 786 male employees returned to work, based on physicians’ “RTW” certificates. Two hundred forty-seven employees never returned to work. The RTW rate in this study was 76.1%. As shown in Table 1, the mean age of the subjects was 52.1 years. With regard to cancer site, survivors with “Gastric” cancers were the most prevalent (ICD-10: C16, *n* = 234), followed by “Intestinal” cancers (C17–C21, *n* = 114), including small intestine cancer (*n* = 4), colon cancer (*n* = 51), and rectal or anal cancer (*n* = 59). The third most prevalent was “Lung” cancers (C33–C34, *n* = 104). The median duration of the first sick leave among all subjects was 93 days, and the RTW employees in the “Blood” cancer groups required a longer period of sick leave than others (198 days, almost 6.5 months). Of the 786 male cancer survivors, 300 had recurrent sick leave due to cancer, including death, and 76 cancer survivors resigned from their companies after RTW. The median duration of work after RTW among all cancer survivors was 4.5 years.

**Table 1.** Basic characteristics of the subjects in this study

Cancer site	Total *N*	Mean (SD) age, years	Median duration of the first sick leave, days	Recurrent sick leave (*N* = 300)	Resigned (*N* = 76)	Median duration of work after RTW, years
Gastric	234	52.9 (5.1)	65	56	33	10.9
Intestinal	114	51.6 (6.0)	81	43	4	8.3
Lung	104	53.6 (4.8)	97	55	25	0.9
Male genital	66	52.4 (7.4)	94	13	2	—
Blood	61	48.7 (8.0)	198	32	1	3.8
Hepatic, pancreatic	45	54.4 (4.9)	79	26	4	0.9
Urinary	43	52.3 (5.7)	84	20	1	2.8
Esophageal	40	53.6 (3.9)	94	21	5	1.5
Other	79	50.1 (7.7)	114	34	1	7.1

Total	786	52.1 (6.1)	93	300	76	4.5

RTW, return to work; SD, standard deviation.

### Work continuance rates after RTW among male cancer survivors

**Figure 1.**
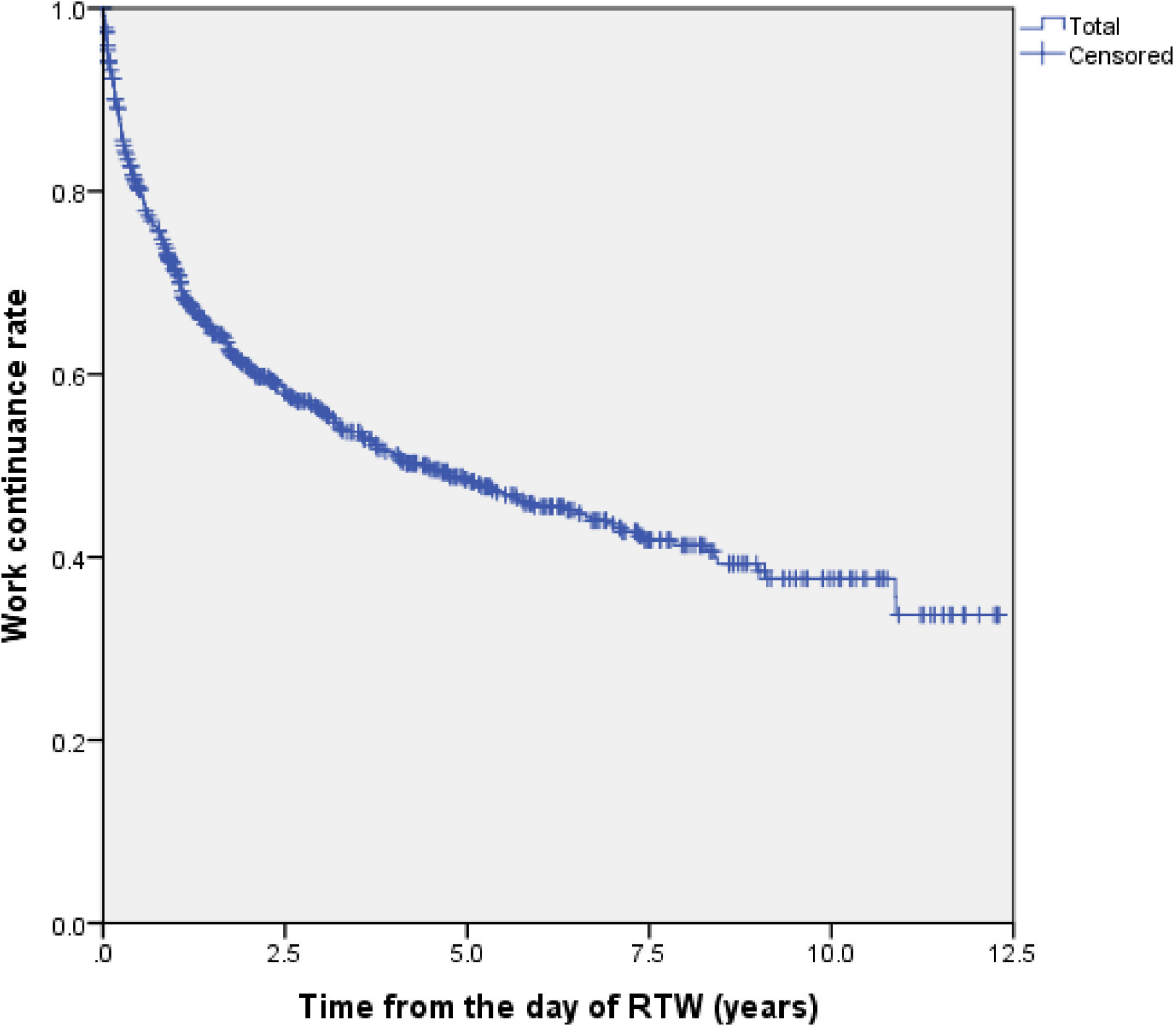
Work continuance rates from the day of RTW using Kaplan-Meier survival analysis. RTW, return to work.

**Figure 2.**
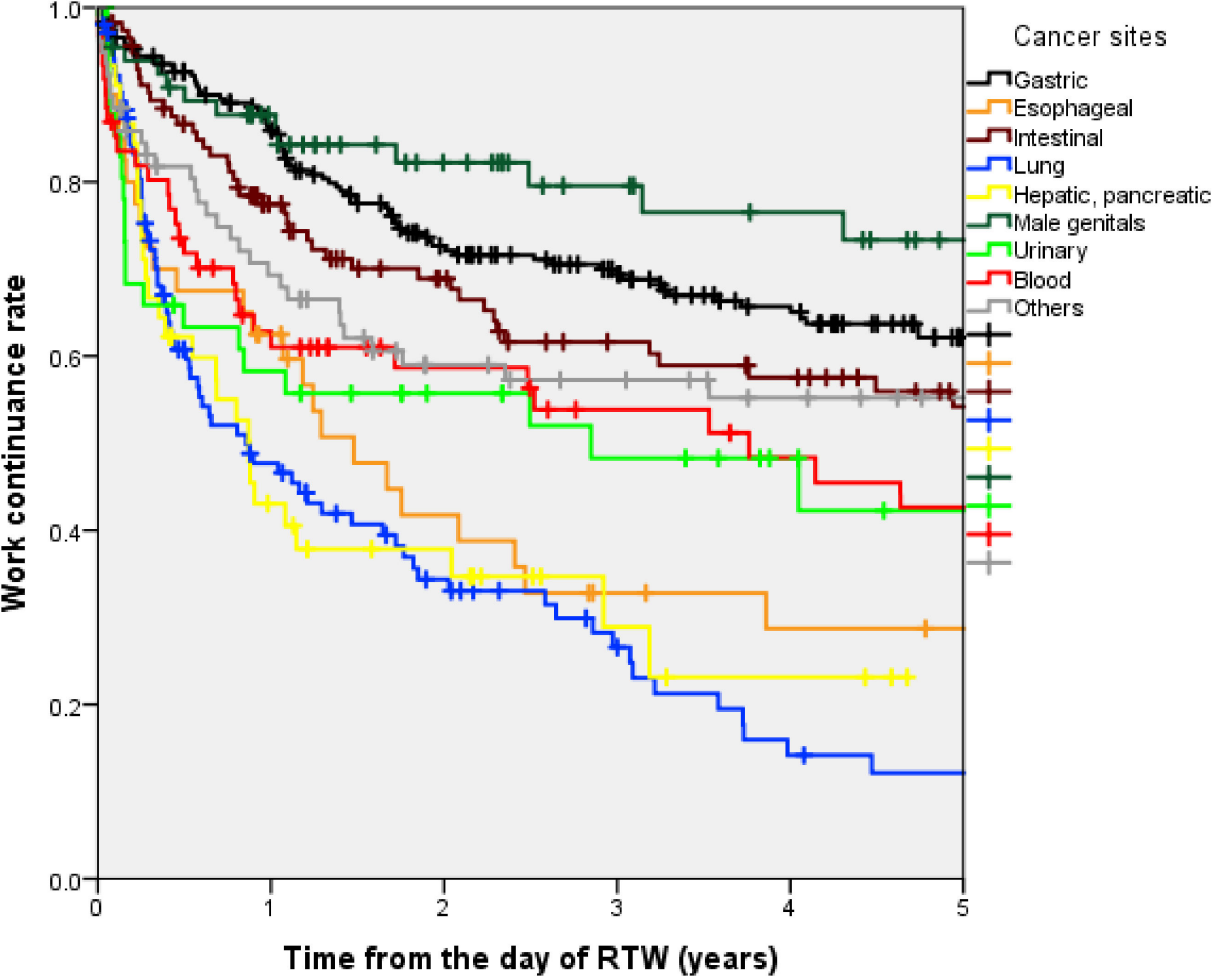
Work continuance rate from the day of RTW, stratified by cancer sites. RTW, return to work.

Using the Kaplan-Meier survival estimate, work continuance rates among all the subjects were 80.1% at 6 months, 71.2% at 1 year, 60.9% at 2 years, 56.1% at 3 years, 51.4% at 4 years, and 48.5% at 5 years after the RTW date, as shown in Figure 1. Up to 5 years after the RTW date, almost half of the employees (48.5%) continued to work without recurrent sick leave or resignation. As shown in Figure 2, there was a steep decrease in work continuance rates during the first year after RTW.

As shown in Figure 2, there were considerable differences in the range of work continuance rates according to cancer site. The “Lung” cancer group had the lowest work continuance rate at 6 months, 1 year, 2 years, 3 years, and 5 years after the RTW date: 60.7%, 47.7%, 34.4%, 26.6%, and 12.1%, respectively. The second lowest work continuance rate was reported in the “Hepatic, Pancreatic” cancer group: 62.2% at 6 months, 43.1% at 1 year, 37.8% at 2 years, 28.9% at 3 years, and 23.1% at 5 years. The highest work continuance rate was reported in the “Male genital” cancer group: 90.8% at 6 months, 87.7% at 1 year, 82.2% at 2 years, 79.6% at 3 years, and 73.3% at 5 years.

### Predictors for the failure of work sustainability after RTW among cancer survivors

Table 2 shows the predictors for the time to recurrent sick leave or resignation, by age, duration of the first sick leave, cancer site, calendar year, manager, and job title. According to the multivariate analysis including all variables, subjects in the >57 year-old groups were at greater risk of “not continuing work” than the <48 year-old (reference) age groups (hazard ratio 1.71; 95% confidence interval, 1.16–2.51). Subjects with >181 days of sick leave had more difficulties working continuously than those with <60 (hazard ratio 2.32; 95% confidence interval, 1.54–3.49). The “Esophageal”, “Lung”, “Hepatic, pancreatic”, and “Urinary” cancer groups were at greater risk of recurrent sick leave or resignation than the “Gastric” cancer group. Managers had more difficulties working continuously after RTW than non-managers (hazard ratio 1.82; 95% confidence interval, 1.19–2.77). Manual workers had a longer time to recurrent sickness leave or resignation than non-manual workers (hazard ratio 0.74; 95% confidence interval, 0.58–0.95).

**Table 2.** Cox regression model for the time to work sustainability failure

Variables	Categories	Single variable analysis		Multivariable analysis	
	
		HR (95% CI)	Trend *P*	HR (95% CI)	Trend *P*
Age, years			<0.01		0.05
	<48 (ref)	1		1	
	49–52	1.30 (0.96–1.77)		1.19 (0.84–1.67)	
	53–56	1.36 (1.02–1.81)		1.32 (0.96–1.82)	
	>57	1.68 (1.22–2.31)		1.71 (1.16–2.51)	

Duration of sick leave, days			<0.01		<0.01
	<60 (ref)	1		1	
	61–180	1.52 (1.22–1.90)		1.46 (1.15–1.87)	
	>181	2.17 (1.60–2.96)		2.32 (1.54–3.49)	

Cancer sites					
	Gastric (ref)	1		1	
	Intestinal	1.22 (0.86–1.73)		1.12 (0.76–1.66)	
	Lung	3.75 (2.76–5.09)		3.31 (2.38–4.62)	
	Male genital	0.65 (0.37–1.16)		0.52 (0.29–0.92)	
	Blood	1.85 (1.24–2.77)		1.15 (0.70–1.88)	
	Hepatic, pancreatic	3.43 (2.26–5.22)		2.74 (1.72–4.36)	
	Urinary	1.91 (1.19–3.08)		1.90 (1.13–3.18)	
	Esophageal	2.58 (1.66–4.00)		2.30 (1.44–3.67)	

Calendar year (year of sick leave)					
	2000–2003 (ref)	1		1	
	2004–2007	0.93 (0.73–1.17)		0.93 (0.72–1.20)	
	2008–2011	1.22 (0.93–1.59)		1.01 (0.74–1.37)	

Company size					
	≥1000 employees (ref)	1		1	
	<1000 employees	0.92 (0.66–1.27)		0.81 (0.57–1.15)	

Manager/non-manager					
	Non-manager (ref)	1		1	
	Manager	1.87 (1.26–2.77)		1.82 (1.19–2.77)	

Job title					
	Desk worker (ref)	1		1	
	Manual worker	0.76 (0.60–0.97)		0.74 (0.58–0.95)	

CI, confidence interval; HR, hazard ratio.

^a^Adjustment for age, duration of sick leave, cancer sites, calendar year, company size, manager/non-manager, and job title.

## DISCUSSION

To our knowledge, this is the first workforce-based cohort study investigating the work continuance rate after RTW among male cancer survivors. The present study clarified that almost half of male cancer survivors continued to work for 5 years after RTW, as we hypothesized. The work continuance rate depended significantly on cancer site.

The present study showed that cancer site was closely associated with time to failure of work sustainability (eg, recurrent sick leave and resignation). The present study also showed that, by comparing work continuance rates, survivors of lung, hepatic, pancreatic, or esophageal cancers might have greater difficulty maintaining employment while receiving cancer treatment. However, gastric and male genital cancer survivors were more likely to continue working while receiving cancer treatment.

The Kaplan-Meier curve in this cohort showed that the incidence of work disability (eg, recurrent sick leave or resignation) decreased in the years following RTW; work disability was most frequent in the first year, followed by the second year. Of all the subjects who experienced recurrent sick leave or resigned, 38.6% did so within 6 months after RTW, while 55.9% did so within 1 year. The Kaplan-Meier curves of work continuance rate for different cancer sites seemed to plateau, which was similar to the curve of recurrent sick leave due to depression reported in previous studies.^23^ Based on the results of this study, careful support for cancer survivors should be recommended for 5 years.

According to the results of Cox regression analysis, the present study showed that older subjects (>57 years old) were at greater risk of work disability than younger subjects (<48 years old). There were two reasons for this. Older cancer survivors might resign more frequently than younger survivors. Cancer-related fatigue, which was known as one of the most influential inhibitors of work sustainability, could affect the elderly more than the young.^8^ There was a statistically significant difference between work continuance rate and the years of sick leave due to cancer, whereas the conditional 5-year survival rate for most cancer sites increased with increasing years in Japan.^24^

Subjects who had a longer duration (>181 days) of the first sick leave due to cancer had a greater risk of work disability than the reference population (<60 days). Previous studies have found that cancer site, length of sick leave, and treatment modality (eg, chemotherapy) could be powerful predictors of work sustainability during and after cancer treatment.^15^

To the best of our knowledge, there have been no other longitudinal studies investigating the work continuance rate after the first sick leave due to cancer. De Boer reported that the unemployment rate due to disability among cancer survivors was higher (by almost three times) than controls.^25^ Short et al reported that 9% of cancer survivors resigned from their jobs within a period of 4 years because of cancer-related factors.^15^ Feuerstein et al reported that work circumstances, such as superior and coworker attitudes, physical job demands, organizational policies, and procedures, can strongly affect a cancer survivor’s work outcome.^16^ There have been various types of studies on cancer survivors with differences in study setting, design, and methodology.^26^^–^^28^

In general, maintaining work sustainability after RTW can be challenging, with three types of inhibitors of work sustainability: disease-related inhibitors, such as chemotherapy; physical inhibitors, such as cancer-related fatigue, nausea, diarrhea, and pain; and work-related inhibitors, including commuting and difficulties with colleagues and superiors.^8^^,^^13^ It can be stressful for cancer survivors to reveal their diseases and symptoms to superiors and co-workers and often leads to poor communication between cancer survivors and their employers.^29^

Employment outcomes can be improved with better health care and supportive occupational services aimed at better management of symptoms, rehabilitation, and accommodation of disabilities.^30^ Occupational health professionals (eg, occupational physicians and occupational health nurses) can assist cancer survivors with such distress, in the sense of helping patients to regain the roles that they held in society before being diagnosed with cancer, by improving communication, securing a partial RTW, and decreasing workload.^31^^–^^33^ In the present study, the RTW support system may be the reason work sustainability rates after RTW seemed to be very high. Large-scale companies provide a part-time work system, work accommodation, and OP interviews. We speculate that many small- and medium-sized enterprises in Japan have lower work sustainability rates than the large-scale companies examined in this study. We have started to collect the data of the cancer survivors who returned to work in small- and medium-sized companies in order to compare the work sustainability rates between companies of different sizes.

### Strengths and limitations

One of the strengths of the present study is that it was the first large-scale data analysis of Japanese employees who returned to work after a period of sick leave due to cancer. Second, the maximum follow-up period was 12.3 years, which is a sufficient follow-up period to evaluate the work sustainability of cancer survivors. Third, we used an objective measurement of sick leave: the present study was based on physicians’ certificates. This study seems to have higher validity and reliability than other studies, which were based on self-administered questionnaires.

As for the limitations of this study, we advise caution when interpreting the results of this study. First, in this study, no detailed medical information on the subjects was available, such as the stage of cancer and type of treatment (eg, surgery, chemotherapy, or radiation therapy), cancer-related side effects, and cancer-related symptoms. In particular, Feuerstein et al pointed out that cancer-related fatigue reduced the quality of life of cancer survivors.^8^ In future studies, work sustainability after RTW should be evaluated with respect to clinical medicine, symptomatology, and psycho-oncology. Second, because all subjects were male, caution is necessary when generalizing across the entire workforce based on the present study results. More research is needed to fully investigate work sustainability among female cancer survivors. As the number of breast cancer survivors has increased drastically in Japan, occupational support for female cancer survivors after RTW should be increased. Third, as the data of this study were collected from large-scale companies, forming generalizations for small- and medium-sized enterprises might be difficult. Fourth, as the data of this study were from the day of RTW to the day of recurrent sickness absence, resignation and so on, the work sustainability among cancer survivors could be underestimated. While some employees quit their job after RTW in this study, they could remain a member of the workforce in other companies. Fifth, the number of participants with a missing value for ‘job title’ was 107 (13.6%). The result of sensitivity analysis showed that there were little difference between the multivariable analysis including ‘job title’ or not. Future studies should investigate, in more detail, work-related predictors of work sustainability after RTW.

### Conclusion

Of workers who returned to work after their first episode of leave after cancer, 50% continued to work after 5 years in large-scaled companies. There was a steep decrease in work continuance rates during the first year after RTW, with considerable differences according to cancer site.

## References

[1] Aziz NM Cancer survivorship research: state of knowledge, challenges and opportunities. Acta Oncol. 2007;46:417–432. 10.1080/02841860701367878 17497308

[2] Endo M, Haruyama Y, Takahashi M, Nishiura C, Kojimahara N, Yamaguchi N Returning to work after sick leave due to cancer: a 365-day cohort study of Japanese cancer survivors. J Cancer Surviv. 2016;10(2):320–329. 10.1007/s11764-015-0478-3 26318185 PMC4801999

[3] Pryce J, Munir F, Haslam C Cancer survivorship and work: symptoms, supervisor response, co-worker disclosure and work adjustment. J Occup Rehabil. 2007;17:83–92. 10.1007/s10926-006-9040-5 17318459

[4] Marino P, Teyssier LS, Malavolti L, Le Corroller-Soriano AG Sex differences in the return-to-work process of cancer survivors 2 years after diagnosis: results from a large French population-based sample. J Clin Oncol. 2013;31:1277–1284. 10.1200/JCO.2011.38.5401 23358985

[5] Matsuda A, Matsuda T, Shibata A, Cancer incidence and incidence rates in Japan in 2008: a study of 25 population-based cancer registries for the Monitoring of Cancer Incidence in Japan (MCIJ) project. Jpn J Clin Oncol. 2014;44:388–396. 10.1093/jjco/hyu003 24503029

[6] Ministry of Health Labor W, Japan. Jigyojou ni okeru tiryo to syokugyouseikatu ni okeru ryouritusien ni okeru gaidorain (Guideline for support for therapy and work life in Japanese workforce). 2016. (in Japanese).

[7] Peteet JR Cancer and the meaning of work. Gen Hosp Psychiatry. 2000;22:200–205. 10.1016/S0163-8343(00)00076-1 10880715

[8] Feuerstein M. *Work and Cancer Survivors*. Springer; 2009.

[9] Amir Z, Moran T, Walsh L, Iddenden R, Luker K Return to paid work after cancer: a British experience. J Cancer Surviv. 2007;1:129–136. 10.1007/s11764-007-0021-2 18648953

[10] van Muijen P, Weevers NL, Snels IA, Predictors of return to work and employment in cancer survivors: a systematic review. Eur J Cancer Care (Engl). 2013;22:144–160. 10.1111/ecc.12033 23279195

[11] Amir Z, Neary D, Luker K Cancer survivors’ views of work 3 years post diagnosis: a UK perspective. Eur J Oncol Nurs. 2008;12:190–197. 10.1016/j.ejon.2008.01.006 18342571

[12] Taskila T, Martikainen R, Hietanen P, Lindbohm ML Comparative study of work ability between cancer survivors and their referents. Eur J Cancer. 2007;43:914–920. 10.1016/j.ejca.2007.01.012 17314041

[13] Verbeek J, Spelten E, Kammeijer M, Sprangers M Return to work of cancer survivors: a prospective cohort study into the quality of rehabilitation by occupational physicians. Occup Environ Med. 2003;60:352–357. 10.1136/oem.60.5.352 12709521 PMC1740540

[14] Amir Z, Brocky J Cancer survivorship and employment: epidemiology. Occup Med (Lond). 2009;59:373–377. 10.1093/occmed/kqp086 19692522

[15] Short PF, Vasey JJ, Tunceli K Employment pathways in a large cohort of adult cancer survivors. Cancer. 2005;103:1292–1301. 10.1002/cncr.20912 15700265

[16] Feuerstein M, Todd BL, Moskowitz MC, Work in cancer survivors: a model for practice and research. J Cancer Surviv. 2010;4:415–437. 10.1007/s11764-010-0154-6 20945110

[17] Gudbergsson SB, Fosså SD, Borgeraas E, Dahl AA A comparative study of living conditions in cancer patients who have returned to work after curative treatment. Support Care Cancer. 2006;14:1020–1029. 10.1007/s00520-006-0042-9 16572315

[18] Bradley CJ, Bednarek HL, Neumark D Breast cancer survival, work, and earnings. J Health Econ. 2002;21:757–779. 10.1016/S0167-6296(02)00059-0 12349881

[19] Yabroff KR, Lawrence WF, Clauser S, Davis WW, Brown ML Burden of illness in cancer survivors: findings from a population-based national sample. J Natl Cancer Inst. 2004;96:1322–1330. 10.1093/jnci/djh255 15339970

[20] Taskila-Abrandt T, Pukkala E, Martikainen R, Karjalainen A, Hietanen P Employment status of Finnish cancer patients in 1997. Psychooncology. 2005;14:221–226. 10.1002/pon.838 15386773

[21] Arbowet. Arbowet (Working Conditions Act) http://www.arbo.nl. 1998.

[22] Waddell G, Burton AK Occupational health guidelines for the management of low back pain at work: evidence review. Occup Med (Lond). 2001;51:124–135. 10.1093/occmed/51.2.124 11307688

[23] Endo M, Haruyama Y, Muto T, Yuhara M, Asada K, Kato R Recurrence of sickness absence due to depression after returning to work at a Japanese IT company. Ind Health. 2013;51:165–171. 10.2486/indhealth.2012-0063 23095329

[24] Ito Y, Miyashiro I, Ito H, Long-term survival and conditional survival of cancer patients in Japan using population-based cancer registry data. Cancer Sci. 2014;105:1480–1486. 10.1111/cas.12525 25183551 PMC4462379

[25] de Boer AG, Taskila T, Ojajärvi A, van Dijk FJ, Verbeek JH Cancer survivors and unemployment: a meta-analysis and meta-regression. JAMA. 2009;301:753–762. 10.1001/jama.2009.187 19224752

[26] Roelen CA, Koopmans PC, Groothoff JW, van der Klink JJ, Bültmann U Sickness absence and full return to work after cancer: 2-year follow-up of register data for different cancer sites. Psychooncology. 2011;20:1001–1006. 10.1002/pon.1820 20672244

[27] Murray K, Lam KB, McLoughlin DC, Sadhra SS Predictors of return to work in cancer survivors in the Royal Air Force. J Occup Rehabil. 2015;25:153–159. 10.1007/s10926-014-9516-7 25038986

[28] Hensing G Swedish Council on Technology Assessment in Health Care (SBU). Chapter 4. Methodological aspects in sickness-absence research. Scand J Public Health Suppl. 2004;63:44–48. 10.1080/14034950410021844 15513653

[29] Brown RF, Owens M, Bradley C Employee to employer communication skills: balancing cancer treatment and employment. Psychooncology. 2013;22:426–433. 10.1002/pon.2107 22162192

[30] Steiner JF, Cavender TA, Main DS, Bradley CJ Assessing the impact of cancer on work outcomes: what are the research needs? Cancer. 2004;101:1703–1711. 10.1002/cncr.20564 15386303

[31] Buijs P, van Amstel R, van Dijk F Dutch occupational physicians and general practitioners wish to improve cooperation. Occup Environ Med. 1999;56:709–713. 10.1136/oem.56.10.709 10658552 PMC1757665

[32] Spelten ER, Sprangers MA, Verbeek JH Factors reported to influence the return to work of cancer survivors: a literature review. Psychooncology. 2002;11:124–131. 10.1002/pon.585 11921328

[33] Wynn P, Woodcock K Occupational health referrals for advice on cancer-related sickness absence. Occup Med (Lond). 2011;61:202–204. 10.1093/occmed/kqr012 21525072

